# The effect of storage periods and SPIDES on embryonic mortality, hatching characteristics, and quality of newly hatched chicks in broiler eggs

**DOI:** 10.1007/s11250-023-03547-x

**Published:** 2023-03-27

**Authors:** H M Okasha, G M El-Gendi, K M Eid

**Affiliations:** 1 Animal Production Department, Faculty of Agriculture, Benha University, Moshtohor, 13736 Egypt; 2 Animal Production Research Institute, Agriculture Research Center, Giza, 12618 Egypt

**Keywords:** SPIDES, Hatching window, Chick quality, Incubation

## Abstract

Egg storage duration can affect embryo mortality, hatching characteristics, hatching time, and post-hatch chick quality. In order to assess these effects, the impact of storage duration (5 days, 10 days, 15 days) and short incubation period during egg storage (SPIDES) investigated further 18, 900 eggs of broiler breeder (ROSS 308) in 3 × 2 factorial arrangement design. In the SPIDES treatment, the egg shell temperature was raised from its storage temperature (18 °C) and held at 100 °F for 3.5 h. Storage periods could significantly (*P* < 0.05) influence on embryo mortality (total, early, middle, and late), hatchability of both the total eggs and fertile eggs. The SPIDES treatment had a significant (*P* < 0.05) impact on a lower embryonic death rate and improved egg hatchability. Eggs stored for 5 days and eggs treated with SPIDES significantly (*P* < 0.001) shorten hatching time, batch’s 90% hatching time (T 90% H), mean hatching time (MHT), maximal hatching period (MHP), and hatching window (HW). Chick quality was also determined, whereas storing eggs for 5 days and using the SPIDES treatment resulted in enhanced (*P* < 0.001) chick weight relative to egg weight (CW/EW), activity (AC), and chick quality score (CQS). The residual yolk sac weight (RYSW), unhealed navel (UHN %), and dirty feather (DF%) recorded the lowest (*P* < 0.001) values compared to long storage periods and control group. Finally, stored for 5 days treated by SPIDES positively affected the hatchability characteristics, the shortening hatching time, and the quality of chicks. Regarding the results, it was confirmed that using the SPIDES treatment to prevent the harmful effects of broiler eggs being stored for an extended period of time is a viable option.

## Introduction

In broiler, hatcheries for grandparents and their parents, egg storage is a standard method to organize hatchery operations and predict need (Adriaensen et al., 2022; Brady et al., 2022; Maman and Yildirim, 2022; Özlü et al., 2021a). When an egg is stored in a refrigerator, the temperature is significantly below physiological zero, which Edwards (1902) described as 21 to 27 °C (Funk and Biellier (1944)). At this phase, it is possible that blastodermal cells can go through mitosis (Arora and Kosin (1968)); nonetheless, embryonic activity is halted (Bakst and Gupta, 1997; Fasenko et al., 2001a). Eggs stored in cool environments for up to 7 days hatchability are negligibly affected, according to most experts. On the other side, extended storage times have been connected to a decline in hatchability (Fasenko, 2007; Fasenko et al., 2001b) and the quality of chicks (Tona et al., 2003, 2004) and increasing incubation times (Reijrink et al., 2010). Fasenko (2007) and Hamidu et al. (2010) hypothesized that long-term egg storage stresses out the developing embryo, resulting in increased necrotic and apoptotic cell death and, as a result, developmental deficits and a reduced metabolism. The outcome is that irreversible, sustained harm to the embryo could occur, increasing embryonic mortality and poor chick performance. The embryo will be kept at body temperature during the day as the egg forms in oviduct, and normal embryonic development will take place once the egg is laid. The embryo will have roughly 30,000 cells and be in stages X–XII of gestation. Embryo development normally stops once the eggs are chilled for storage, and if the eggs are stored at a temperature beneath 24 °C (Eyal-Giladi and Kochav, 1976), embryo cells begin to die once eggs have been stored for more than a few days. After 10–12 days of storage, more than half of the cells present at oviposition will have perished (Bakst et al., 2012). The length of storing period besides heat seems to have the biggest effects on albumen quality (Jin et al., 2011; Maman and Yildirim, 2022; Özlü et al., 2021a; Samli et al., 2005; Yimenu et al., 2018). The pH of the albumen is a helpful tool for checking albumin quality fluctuations during storage (Akyurek and Okur, 2009); due to the eggshell’s pores allowing CO_2_ to escape, albumen’s pH rises quickly (alkalinizes) (Grashorn et al., 2016; Özlü et al., 2021a; Uyanga et al., 2020). In order to make up for such decreased hatchability and quality of newly hatched chicks brought on by lengthy storage, several approaches have been devised. In several topical research on eggs retained in breeder facilities for a very long time, “SPIDES” have been investigated in further detail. Damaziak et al. (2021), Dymond et al. (2013), French et al. (2011), Nicholson (2012), Nicholson et al. (2011), Nicholson et al. (2013), and Özlü et al. (2021a), they discovered a favourable effect on chick appearance and hatchability. Nicholson et al. (2013) documented that in SPIDES treatment, the highest outcomes showed whenever the shell temperature was above > 32°C for 2–5 h and maintained at 35 °C for 2–3 h. Özlü et al. (2021a) and Reijrink et al. (2009) hypothesized that fetus is in the pre-gastrula phase of growth at egg collection, and SPIDES treatment with extended storage periods is most advantageous. Özlü et al. (2018), Özlü et al. (2021a), and Pokhrel et al. (2018) they noted that newly laid eggs from younger breeder flocks exhibit earlier embryonic mortalities than eggs from older breeder flocks.

Despite the fact that storage times and SPIDES have each been studied separately, fewer studies have been done in this direction about how these two factors interact to affect incubation traits, the hatching window, and chick quality. This research looked at all three in order to determine how storage times, a short period of incubation during egg storage (SPIDES), and their interactions affected embryonic mortality, hatching traits, and chick quality.

## Materials and methods

### Study design

The trial used a factorial design (3 × 2) with three storage durations (5, 10, and 15 days) separated into two treatments (control and SPIDES) per each storage period.

### Conditions for collecting and storing eggs

The Institutional Animal Care and Use Committee (IACUC) of Benha University gave its approval to all experimental methods. Eggs were collecting and obtained from (ROSS 308) hens at 45 weeks of age. Within the same geographic region, hens received the same diet and management plan. In order to guarantee that eggs produced late the day before were excluded from the collection, eggs were retrieved from nests as soon as the nesting homes’ lights were turned on. Freshly collected eggs from the second collection of the day were kept for one day at 17 °C and 65–70% relative humidity in an egg storage facility on the farm. The following morning, eggs were shipped in a temperature-controlled truck for around 3.5 h to a hatchery (Association of Al-Tanmia for hatching and poultry production). Setter trays were used to preserve eggs for three periods 5, 10, or 15 days at 18 °C comparable relative humidity 75%. While in storage period, either the eggs were left in storage chambers (control) or SPIDES was applied to them on the fourth day of storage in each storage period. SPIDES patterns were used to raise the temperature of eggs from their storage temperature to a 100°F intended for egg shells. This was conducted in a Pas Reform®-Smart machine (Model V6.0 Smart Set TM, Smart Hatch TM) in about 3.5 hours. Hatching eggs were moved from the storage room (18°C) to the setter room, where the temperature of the eggs gradually rose to reach 75.4 °F. Thereafter, hatching eggs fell into the setter, and the time of 3.5 h is calculated from the egg shell temperature reaching 100 °F (random sample of 225 eggs per storage period); a temperature was measured by the Braun ThermoScan device, which had been warming in the setter for about 15 minutes prior to beginning the test. Then, the eggs were taken out of the setter and left to rest in the setter room in order to egg temperature decreased gradually to reach 75.4 °F. After that, the eggs were put back in the same coolers as such control eggs.

### Incubation

A single incubator and hatcher with capacities of 115,200 and 19200 eggs, respectively, were used to incubate a total of 18,900 eggs. 6300 eggs from each storage period were divided into the two groups (control and SPIDES) at random. The hatching eggs which were never a component of the study took up the remaining space in the incubator to ensure even air circulation throughout the eggs. A single-stage incubation program was used (Table 1). The eggs were turned over once every hour up to day 18.5 of incubation. They were then carried to baskets and placed inside a hatcher. The initial temperature setting for the hatcher was 98° F, and over the past three days, it gradually dipped to 97 °F (until day 21). To lessen the effect of any differences in incubation circumstances between treatments which could be brought about by minuscule changes in airflow over through the eggs, the trays representing each storage period and SPIDES applied were dispersed at random distributed across the incubator and hatcher. There were 21 replicates per sub-treatment group, with every 150 egg/plate being treated as a single replicate.

**Table 1:** Applied single-stage incubation program in this study

Incubation		Incubation temperature		Relative humidity	Ventilation
Day	Hours	Set point (°F)	Eggshell temp. (°F)	Set point (%)	Set point (% valve)
1	0–24	100.4	100.0	53	0
2	48	100.2	100.0	53	0
3	72	100.0	100.0	53	0
4	96	99.9	100.0	53	10
5	120	99.9	100.0	53	10
6	144	99.9	100.0	53	10
7	168	99.9	100.0	53	20
8	192	99.8	100.0	53	30
9	216	99.8	100.0	53	40
10	240	99.8	100.0	53	40
11	264	99.7	100.0	53	50
12	288	99.5	100.0	53	50
13	312	99.2	100.0	53	60
14	336	98.8	100.1	53	70
15	360	98.5	100.5	53	70
16	384	98.3	101.0	53	80
17	408	98.0	101.5	53	90
18	432	98.0	101.8	53	100
18.5	444	98.0	101.8	53	100

### Measurements

#### Embryonic mortality and hatchability

On day 10, candling was used to identify defective eggs and embryos dead earlier from 0 to 7 days and which are removed. The opened eggs were then examined macroscopically. After the chicks were taken out of the hatcher, the all remaining eggs were cracked, and a single specialist examined them macroscopically to check for any residual embryonic mortality, which was divided into early (0–7 days), medium (8–17 days), and late (81–21 days) categories. Hatchability is calculated according to the following equation:

$$HFE=\frac{No.\;of\;hached\;chicks}{No.\;of\;fertile\;eggs}\times100$$

#### Hatching time

To observe the hatching procedure, the hatcher was turned off at 510 h. From 480 to 510 h following incubation, the number of chicks that hatched was counted every 2 h. The time of hatching (TH), the time it took to achieve the 90% hatch (T 90% H), the mean hatch time (MHT), the maximal hatching period (MHP), and the hatching window (HW) records were established per each replication. The definition of hatch time was the timing at which 100% of the batch hatched. The hatching time at which the batch’s 90% of eggs hatched was referred as “time took to achieve the 90% hatch”. The sum of all chicks’ hatching times was used to get the average and/or mean of hatching time. The time frame between 30 and 80% of the batch hatching was used to establish the peak or maximal hatching period (Zhong et al., 2018). “Hatch-window” is the time period between the hatching time of the first chick and that of the last chick (Careghi et al., 2005; Zhong et al., 2018).

#### Chick quality

After 510 h of incubation, all of the hatched chicks were examined macroscopically within 4 to 6 h after hatching in order to point out the numerous characteristics that can be linked to good-, moderate-, or poor-quality chicks, adopting the approach shown by Tona et al. (2003). Similarly, residual yolk sac weight (50 chicks/ treatment) was measured according to the method of the previous author. The distance between the beak’s tip and the middle toe’s nail implantation was used to determine the length of the chick (Hill, 2001; Willemsen et al., 2008). The chick weight to egg weight ratio was calculated by using the average weight of each replicate. Additionally, the belly area, activity, and appearance (plumage) are all given an overall of 100 following the inspection process (every 4–6 h).

#### Statistical analysis

Data were analyzed via factorial ANOVA using the GLM procedure in SAS (SAS, 2004). The model was used for statistical analysis of embryonic mortality, hatchability characteristics, hatching time, and chick quality:

## Y_ijk_ = µ + S_i_ + T_j_ + (ST)_ij_ + e_ijk_

where *Y*_*ijk*_ is the *k*^th^ observation; *µ* is the overall mean; *S*_*i*_ is effect of the *i*^th^ storage periods; *T*_*j*_ is the effect of the *j*^th^ SPIDES treatment; *(ST)*_*ij*_ is the interaction between *i*^th^ storage periods and *j*^th^ SPIDES treatment; and *e*_*ijk*_ is tthe experimental error, accordingly zero mean and variance = ${\sigma }^{2}e$. Duncan’s multiple range tests were used to identify differences between the treatment means. *P* < 0.05 was used to evaluate statistical significance unless otherwise stated.

## Results and discussions

### Embryonic mortality and hatching characteristics

The effects of storage periods (ST) and SPIDES on embryonic mortality, hatching performance characteristics, and interaction between them are shown in Table 2. Storage periods, SPIDES, and interactions between them had a significant effect on total embryonic mortality and its different stages, as well as percentage of hatchability of all (HTE) and fertilized eggs (HFE), respectively. Eggs treated with SPIDES and kept for 5 days and their interaction recorded lower total embryonic mortality and different stages of death during incubation (*P* < 0.05). Furthermore, HTE and HFE had significantly higher percentages of hatchability (*P* < 0.05).

**Table 2:** Embryonic mortality and hatchability in response to storage period and SPIDES treatment

Factors	Levels	Total embryonic mortality (%)	Early embryonic mortality (%)	Mid embryonic mortality (%)	Late embryonic mortality (%)	Hatchability of total egg (%)	Hatchability of fertile egg (%)
SP	5 days	5.29^c^	2.35^b^	0.91^b^	2.03^c^	88.66^a^	94.71^a^
	10 days	7. 90^b^	3.64^a^	0.85^b^	3.41^b^	86.66^b^	92.10^b^
	15 days	10.85^a^	4.88^a^	1.55^a^	4.42^a^	83.51^c^	89.15^c^
	SEM	0.179	0.127	0.078	0.126	0.107	0.179
PI	Control	8.74^a^	3.74^b^	1.33^a^	3.67^a^	85.81^b^	91.26^b^
	SPIDES	7.29^b^	4.17^a^	0.90^b^	2.22^b^	86.74^a^	92.71^a^
	SEM	0.146	0.104	0.063	0.103	0.087	0.146
SP × PI	5 days × C	5.58^e^	1.73^d^	1.29^b^	2.56^bc^	88.39^b^	94.42^a^
	5 days × PI	5.00^e^	2.97^c^	0.54^c^	1.49^d^	88.93^a^	95.00^a^
	10 days × C	8.54^c^	5.14^a^	0.83^c^	2.57^bc^	85.86^d^	91.46^c^
	10 days × PI	7.25^d^	4.14^b^	0.86^c^	2.25^c^	87.45^c^	92.75^b^
	15 days × C	12.08^a^	4.35^b^	1.83^a^	5.90^a^	83.17^f^	87.92^e^
	15 days × PI	9.62^b^	5.40^a^	1.31^b^	2.91^b^	83.86^e^	90.38^d^
	SEM	0.254	0.180	0.110	0.178	0.152	0.254
*P* value	SP	0.0001	0.0001	0.0001	0.0001	0.0001	0.0001
	PI	0.0038	0.0038	0.0001	0.0001	0.0001	0.0001
	SP × PI	0.0001	0.0001	0.0028	0.0001	0.0013	0.0014

The data represents the average of 21 replicates

Mean having similar letters in each column within each effect are not significantly different

Abbreviations: *SP*, storage periods (5, 10, 15 days); *PI*, pre-incubation heat treatment (control and SPIDES); *SPIDES*, short period of incubation during egg storage

The number of early, medium, late, and total embryos that perished rose when eggs were kept in storage longer without receiving SPIDES treatment. An association between storage time and SPIDES for these two factors was indeed found (Okur et al., 2018; Özlü et al., 2018, 2021a); additionally, they noticed a higher risk of embryonic death with long-term egg preservation. Tona et al. (2004) ascribed the longer-term storage of eggs’ reduced hatchability to the albumen’s inferior quality than that of freshly oviposited eggs. Due to the proximity of the blastoderm to the albumen, alterations in the viscosity or pH of the albumen may have a significant impact on the viability of the embryo during its first developmental stages (Maman and Yildirim, 2022; Özlü et al., 2021a). Alternately, the higher embryonic mortality may be due to variations in the fetal developmental phase at oviposition. Eggs saved for 5 days had a higher percentage of hatchability than eggs kept for 15 days. The outcomes are in line with those of Damaziak et al. (2021); Pokhrel et al. (2018) discovered that hatchability was shown to be greater after 7 days of storage compared to when it was at 0 days, and it decreased with longer storage intervals (21 to 28 days). This may be due to long-term storage, which raises albumen pH and lowers albumen elevation and thickness. Brake et al. (1997) noted that it is likely that albumen liquefied makes it easier for nutrients to go from the albumen to the blastoderm Meuer and Baumann (1988) and through the albumen, it can lower confrontation to the diffusion of gases. Benton and Brake (1996) noticed that in absent egg storage, O_2_ could not be sufficient to sustain the early chick embryo’s metabolic demands, and when incubation begins before liquefaction, as a result, mortality may rise. During the earliest phases of incubation, 4–7 days of hibernation are beneficial for fetal livability (Asmundson, 1947; Brake et al., 1997; Mayes and Takeballi, 1984). Several authors (Pokhrel et al., 2018; Taha et al., 2019; Uyanga et al., 2020; Yalcin et al., 2017) have claimed that extended storing negatively affects hatchability. Modifications in the morphology of the blastoderm provide some explanation (Bakst et al., 2012; Hamidu et al., 2010; Uyanga et al., 2020) and enhanced apoptosis and necrosis of cells (Bakst and Gupta, 1997) during storage. In fact, the total number of blastodermal cells declines as storage time increases as noted by Cai et al. (2019) and Uyanga et al. (2020). Bakst et al. (2012) discovered that kept turkey eggs for 5 days changed the appearance of blastoderm on both a macroscopic and microscopic level; blastoderms appeared to be slightly asymmetrical and larger.

In the present research, due to an improvement in hatchability of 0.93% and 1.45% for the total egg set and fertile egg, respectively, total embryonic mortality in general was lowered (*P* < 0.05) in eggs treated with SPIDES compared to control eggs. Accordingly, Maman and Yildirim (2022), Özlü et al. (2021a), Silva et al. (2008), and Tag EL-Din et al. (2017) indicated that 6 h of warming eggs before storing them for 14 days boosted hatchability and decreased late embryonic mortality. Furthermore, hatchability was enhanced with a heat treatment that included four 4-h pre-incubations spaced 4–5 days apart over a period of 3 weeks of storage at 16 °C mainly by reducing late embryo mortality rather than early death (Dymond et al., 2013). Numerous additional authors, on the other hand, have shown that warming therapy before incubation lowers early mortality (0–7 days) (Fasenko et al., 2001b; Gucbilmez et al., 2013; Nicholson et al., 2013; Reijrink et al., 2010, 2009) and late embryo mortality especially in comparison to unheated eggs (Abdel-Halim et al., 2015; Ebeid et al., 2017; Gharib, 2013).

### Hatching time

The variation in hatching time for both storage periods (SP) and SPIDES treatment is displayed in (Table 3). The brief duration of storage (5 days) and SPIDES treatment was found to give the first hatching consistently compared to prolonged storage periods and control eggs. The time of hatching, the time it took to achieve 90% hatch, the mean hatch time, the maximal hatching period, and the hatching window recorded shorter times in the 5-day storage period and eggs treated with SPIDES than in the other storage periods and un-treated eggs (*P* < 0.01). Only during the maximal hatching period was a significant interaction (*P* = 0.002) found between treatments. The time of hatching, the time it took to achieve 90% hatch, the mean hatch time, the maximal hatching period, and the hatching window (HW) were not as long in eggs stored for 5 days and treated with SPIDES compared to other interactions applied.

**Table 3:** Characteristics linked to incubation time (h) in response to storage period and SPIDES

Factors		TH^1^	T 90% H^2^	MHT^3^	MHP^4^	HW^5^
SP	5 days	504.52^c^	498.45^b^	492.26^c^	8.94^c^	24.52^c^
	10 days	507.00^b^	500.05^b^	493.50^b^	9.89^b^	27.00^b^
	15 days	509.00^a^	503.05^a^	494.50^a^	10.87^a^	29.00^a^
	SEM	0.320	0.591	0.160	0.010	0.320
PI	Control	507.68^a^	502.00^a^	493.84^a^	10.44^a^	27.68^a^
	SPIDES	506.00^b^	499.03^b^	493.00^b^	9.36^b^	26.00^c^
	SEM	0.261	0.482	0.130	0.008	0.261
SP × PI	5 days × C	505.05	500.00	492.52	9.47^d^	25.05
	5 days × PI	504.00	496.90	492.00	8.42^f^	24.00
	10 days × C	508.00	502.00	494.00	10.42^b^	28.00
	10 days × PI	506.00	498.10	493.00	9.37^e^	26.00
	15 days × C	510.00	504.00	495.00	11.45^a^	30.00
	15 days × PI	508.00	502.10	494.00	10.30^c^	28.00
	SEM	0.452	0.836	0.226	0.014	0.452
*P* value	SP	< 0.001	< 0.001	< 0.001	< 0.001	< 0.001
	PI	< 0.001	< 0.001	< 0.001	< 0.001	< 0.001
	SP × PI	0.48	0.49	0.48	0.002	0.48

The data represents the average of 21 replicates

Mean having similar letters in each column within each effect are not significantly different

Abbreviations: *SP*, storage periods (5, 10, 15 days); *PI*, pre-incubation heat treatment (control and SPIDES); *SPIDES*, short period of incubation during egg storage

^1^(TH) described as the hatching time at which all of the eggs in the batch hatched

^2^(T 90% H) was described as the batch's 90% hatching time

^3^(MHT) was defined as the total hatching time of all chicks/the total number of chicks

^4^(MHP) was defined as the time frame between 30 to 80% of the batch hatching

^5^(HW) was calculated by subtracting the hatching time of the last chick from that of the first chick

When investigating the circumstances under which mass egg incubation is occurring in hatcheries, currently, it is obvious that getting the best hatching synchronization and shortening the gap between the first and last chicks to hatch outweighs the length of incubation period. Except for species-specific differences, it has been demonstrated that the mother’s age, the genetic makeup, and the weight of the eggs, as well as the amount of time and the environment in which they were kept before incubation, can all affect how long it takes to hatch an egg (Abdel-Halim et al., 2015; Fasenko, 2007; Ruiz and Lunam, 2002; Vieira et al., 2005). Now that each of these elements is considered in industrial hatcheries, with the intention of treating all eggs during storage similarly and hatching them at the same time using the same reproductive flocks (van de Ven, 2012), however, Tong et al. (2013) detected that hatch window ranges from 24 to 48 h. Consequently, in contrast to precocial birds’ natural development, which takes 3 to 24 h and has at least 3rd eggs in its clutch (Eichholz and Towery, 2010), the reason for this discrepancy is that the insignificant small number of eggs in a normal clutch is in comparison with hundreds of thousands of eggs placed in a hatching device. Also, because the birds take a few days to lay their eggs before starting to incubate them, the last egg in the clutch acts as a signal to begin the hatching process. According to the findings, the amount of time the eggs were stored affected the hatching time, the average hatch time, the peak of the hatching period, and the hatch window. It also affected the time it took for 90% of the eggs to hatch.

In the current study, eggs treated by SPIDES recorded a short average of hatch time, the time it took for 90% of the eggs to hatch, the mean hatching time, and the maximal hatching period, and the first chicks were recorded in 480 h of incubation. The outcomes are consistent with those of Damaziak et al. (2018) and Damaziak et al. (2021) who stated that the first chicks were observed in the first SPIDES group after 477 h and in the second SPIDES group after 479 h. However, the hatching window for the first SPIDES group was relatively long, lasting up to 21 h, whereas it was only 13 h long for the second SPIDES group. Longer egg holding duration was found in the research done by Dymond et al. (2013) and Nasri et al. (2017), and this may be the reason even in the “SPIDES” group, the scientists were able to extend the incubation period by a significant amount: 499–508 h. Also, after 21 days of cold storage, they were able to reduce the pre-incubation heating time to 6 or 12 h. Reijrink et al. (2010) also found a decrease in incubation time utilizing 24 h (PI) throughout 14 days of cold storage. Abdel-Halim et al. (2015) and Damaziak et al. (2018) conceived that the study’s 8 h (PI) in 12-day storage duration was too brief to have a major effect on incubation time reduction and hatching synchronization. Results in this study may be attributed to the temperature of 100 °F that was used for 3.5 hours in the SPIDES treatment, which had a significant effect on reducing incubation time and coordinating hatching.

### Chick quality

Evaluation of the chicks’ quality in accordance with the approach of Table 4 is founded on the classification performed under commercial settings by hatchery staff (Tona et al., 2003). Typically, the highest-graded chicks were those that emerged from eggs that had been stored for 5 days and eggs treated with SPIDES. In both analyses, SPIDES during egg storage had a significant (*P* = 0.001) impact on the quality of chicks for eggs kept at 10 and 15 days. Also, an interaction (*P* < 0.05) was found in residual yolk sac weight (RYSW), chick weight relative to egg weight (CW/EW), and chick quality score (CQS) due to the effect of treatments applied.

**Table 4:** Chick quality in relation to storage period and SPIDES

Factors	Levels	RYSW	CHL	CW/EW	AC	UHN	DF	CQS
SP	5 days	2.66^c^	18.89	67.19^a^	92.25^a^	4.62^c^	1.73^c^	96.05^a^
	10 days	3.47^b^	19.48	67.00^b^	91.05^b^	5.75^b^	2.39^b^	92.11^b^
	15 days	4.61^a^	19.19	67.09^ab^	90.35^c^	6.40^a^	2.94^a^	88.16^c^
	SEM	0.0249	0.0496	0.0517	0.0935	0.0852	0.0092	0.00924
PI	Control	3.86^a^	19.02	67.08	90.05^b^	6.10^a^	2.43^a^	88.63^b^
	SPIDES	3.29^b^	19.35	67.11	92.38^a^	5.08^b^	2.27^b^	95.58^a^
	SEM	0.0209	0.0405	0.0422	0.0763	0.0696	0.0075	0.0754
ST × PI	5 days × C	2.90^e^	18.77	67.29^a^	91.05	5.20	1.81	94.31^c^
	5 days × PI	2.42^f^	19.01	67.10^abc^	93.45	4.05	1.65	97.79^a^
	10 days × C	3.76^c^	19.28	66.91^c^	90.05	6.25	2.48	88.64^e^
	10 days × PI	3.18^d^	19.68	67.08^abc^	92.05	5.25	2.30	95.58^b^
	15 days × C	4.94^a^	19.01	67.03^bc^	89.05	6.85	3.02	82.95^f^
	15 days × PI	4.28^b^	19.38	67.15^ab^	91.65	5.95	2.86	93.37^d^
	SEM	0.0352	0.0702	0.0731	0.0132	0.1205	0.0130	0.0130
*P* value	SP	0.001	0.001	0.03	0.001	0.001	0.001	0.001
	PI	0.001	0.001	0.60	0.001	0.001	0.001	0.001
	SP × PI	0.04	0.48	0.03	0.07	0.55	0.69	0.001

The data represents the average of 21 replicates

Mean having similar letters in each column within each effect are not significantly different Abbreviations: *SP*, storage periods (5, 10, 15 days); *PI*, pre-incubation heat treatment (control and SPIDES); *SPIDES*, short period of incubation during egg storage; *RYSW*, residual yolk sac weight; *CHL*, chick length; *CW/EW*, chick weight relative to egg weight; *AC*, activity (%); *UHN*, unhealed navel (%); *DF*, dirty feather (%); *CQS*, chick quality score

The adverse effects of lengthily egg storage on the health and/or quality of the chicks can be due to the release of carbon dioxide via an eggshell’s pores, where an albumen’s alkalinity quickly increases (Uyanga et al. (2020)); this results in the loss of some germinal disc cells, which may be linked to growth factors like as satellite cells. Skeletal muscle cells have satellite cells under their basal lamina, and these cells have the ability to undergo mitosis; during mitotic divisions, newly created cells merge with muscle fibers already present, increasing their diameter (Zhang et al., 2022). Additionally, the weight loss of the embryo may be to blame. This is an indicator of a lower-quality embryo, which may have an effect on the quality of the hatch (Hamidu et al., 2011). Traditionally, the development speed, hatchability, and quality of broiler egg embryos held for 14 days compared to eggs stored for 4 days were inferior (Fasenko et al., 2001b; Maman and Yildirim, 2022; Özlü et al., 2021a). In another researches of Fasenko (2001) and Christensen et al. (2001), they demonstrated that chicks from eggs that had been kept for a longer period of time had weaker metabolic rates than those from eggs that had been kept for a shorter duration of time, as well as delayed growth of the heart and liver and a drop in relative lung weight, which resulted in poor chick quality (Özlü et al., 2021b; Yalçin and Siegel, 2003). It is necessary to develop strategies that allow for the preservation of eggs for significantly longer periods of time with minimal loss in hatchability and chick quality, consequently enhancing commercial hatchery productivity.

The current investigation revealed that in the SPIDES, treated eggs had a beneficial on chick quality in contrast to the control eggs. Results are in the trend with Ebeid et al. (2017), Gharib (2013), and Maman and Yildirim (2022); they suggested that pre-incubation heating applied for 6 or 8 h increased chicks of first grade and decreased chicks of second grade compared to non-heated controls. As previously reported prolonged storage, the incubation period lengthens (Dymond et al., 2013; Reijrink et al., 2010). Nicholson (2012) noticed that some live chicks are turned away upon take-off because they hatch too late. Furthermore, when compared to early- and middle-hatched chicks, the proportion of second grade chicks can be significantly greater (*P* ˃ 0.05) and live performance lower in late-hatched chicks (Özlü, 2016; Özlü et al., 2018). Some of the chick quality improvement by SPIDES in short storage period for 5 days have been caused by a shorter incubation period and improved capability of an embryo to consume yolk during incubation.

## Conclusions

It can be concluded that both storage periods and SPIDES treatment affect embryonic mortality, hatching characteristics, hatching time, and chick quality. However, an interaction between egg storage and SPIDES treatment was found. Finally, the lowest embryonic mortality, the highest hatchability percentage, the shorter hatching time, and the better chick quality were observed in eggs stored for 5 days and give SPIDES treatment. According to the findings of this study, SPIDES appeared to have independent mechanisms of action and an additive and positive effect in alleviating the effects of longer storage periods on embryonic mortality, hatching characteristics, hatching time, and chick quality.

## Data Availability

The corresponding author can provide the data sets at reasonable request.
